# LncRNA HOTAIR/miR‐613/c‐met axis modulated epithelial‐mesenchymal transition of retinoblastoma cells

**DOI:** 10.1111/jcmm.13796

**Published:** 2018-07-20

**Authors:** Ge Yang, Yang Fu, Xiaoyan Lu, Menghua Wang, Hongtao Dong, Qiuming Li

**Affiliations:** ^1^ Department of Ophthalmology The First Affiliated Hospital of Zhengzhou University Zhengzhou City China; ^2^ Department of General Surgery The First Affiliated Hospital of Zhengzhou University Zhengzhou City China

**Keywords:** c‐met, epithelial‐mesenchymal transition, lncRNA HOTAIR, miR‐613, retinoblastoma

## Abstract

Since lncRNAs could modulate neoplastic development by modulating downstream miRNAs and genes, this study was carried out to figure out the synthetic contribution of HOTAIR, miR‐613 and c‐met to viability, apoptosis and proliferation of retinoblastoma cells. Totally 276 retinoblastoma tissues and tumour‐adjacent tissues were collected, and human retinoblastoma cell lines (ie, Y79, HXO‐Rb44, SO‐Rb50 and WERI‐RB1) were also gathered. Moreover, transfections of pcDNA3.1‐HOTAIR, si‐HOTAIR, miR‐613 mimic, miR‐613 inhibitor, pcDNA3.1/c‐met were performed to evaluate the influence of HOTAIR, miR‐613 and c‐met on viability, apoptosis and epithelial‐mesenchymal transition (EMT) of retinoblastoma cells. Dual‐luciferase reporter gene assay was also arranged to confirm the targeted relationship between HOTAIR and miR‐613, as well as between miR‐613 and c‐met. Consequently, up‐regulated HOTAIR and down‐regulated miR‐613 expressions displayed associations with poor survival status of retinoblastoma patients (*P* < 0.05). Besides, inhibited HOTAIR and promoted miR‐613 elevated E‐cadherin expression, yet decreased Snail and Vimentin expressions (*P* < 0.05). Simultaneously, cell proliferation and cell viability were also less‐motivated (*P* < 0.05). Nonetheless, c‐met prohibited the functioning of miR‐613, resulting in promoted cell proliferation and viability, along with inhibited cell apoptosis (*P* < 0.05). Finally, HOTAIR was verified to directly target miR‐613, and c‐met was the direct target gene of miR‐613 (*P* < 0.05). In conclusion, the role of lncRNA HOTAIR/miR‐613/c‐met signalling axis in modulating retinoblastoma cells’ viability, apoptosis and expressions of EMT‐specific proteins might provide evidences for developing appropriate diagnostic and treatment strategies for retinoblastoma.

## 1 INTRODUCTION

Retinoblastoma, a primary ocular malignant tumour, is originated from infants’ retinal embryonic nuclear layer cells with a worldwide incidence of 0.005%.^1^ Currently, since most children have entered late‐stage retinoblastoma that represented high chance of ocular extraction, early diagnosis and treatment for retinoblastoma appeared to be quite critical.^2^, ^3^ To save children's visual function,^4^ diverse treatment strategies have been developed for varying degrees of retinoblastoma.^5^ Among them, chemotherapy combined with adjuvant therapy has gradually become the preferred treatment, although radiotherapy could cause children's facial deformity and relapse of retinoblastoma.^6^ More than that, the confined efficacies of the therapies also necessitated exploration of novel treatment biomarkers.

Long non‐coding RNAs (lncRNAs) have been demonstrated to matter much in the aetiology of glioma, breast cancer, hepatocellular carcinoma, prostate cancer and retinoblastoma.^7^, ^8^, ^9^, ^10^ Mounting evidences also indicated that lncRNAs could serve as proto‐oncogenes or anti‐oncogenes by regulating tumour‐related genes or pathways.^11^, ^12^ Of note, a microarray analysis confirmed that lncRNAs HOX antisense intergenic RNA (HOAIR), CCAT1 and HIF1A‐AS1 were over‐expressed within retinoblastoma tissues, yet lncRNAs MIR31HG, BCAR4 and H193 were evidently under‐expressed within retinoblastoma tissues.^13^ Among them, HOTAIR was identified to be a cis‐acting parameter within the HOXC loca,^14^, ^15^, ^16^ and its carcinogenicity has been authenticated within breast cancer, liver cancer, colorectal cancer, oesophageal squamous cancer, pancreatic cancer, non‐small cell lung cancer and cervical cancer.^17^, ^18^, ^19^, ^20^, ^21^, ^22^, ^23^, ^24^ In addition, HOAIR was indicated as a potential therapeutic biomarker for retinoblastoma, and silencing of HOTAIR could attenuated proliferation, migration and invasion of retinoblastoma cells were determined after.^25^

One vital mechanism underlying the involvement of HOTAIR in regulating tumour cell proliferation was to modify expressions of relevant miRNAs by acting as competitively endogenous RNAs. For example, HOTAIR could bind to inter‐cellular miR‐331‐3p and miR‐124 based on its characteristic RNA sponging, thereby up‐regulating cancer cells’ capacity to reproduce.^26^ The sponging relationship between HOTAIR and miR‐613 was also manifested within carcinomas, such as non‐small cell lung cancer and pancreatic cancer.^27^, ^28^ The miR‐613 herein was suggested as a suppressor for retinoblastoma formation by targeting E2F5.^29^ However, whether HOTAIR could sponge miR‐613 to modulate osteosarcoma progression remained inconclusive.

Besides, miR‐613 was estimated to prohibit osteosarcoma development by directly targeting c‐met,^30^ which was mainly expressed within epithelial cells, vascular and lymphatic endothelial cells, hematopoietic cells and pericytes. Knockout of c‐met within mice models could give rise to embryonic death, and c‐met was implied to advance embryogenesis through curbing epithelial‐mesenchymal transition (EMT) process of muscle‐derived stem cells, as well as development of neuronal precursors, hepatic tissues and placenta tissues.^31^ Nonetheless, hardly any investigations have pointed out whether HOAIR, miR‐613 and c‐met would cooperate to alter the EMT process of retinoblastoma.

Therefore, this study was aimed to explore if HOAIR sponging miR‐613 might control proliferation, apoptosis and EMT process of retinoblastoma cells through mediating c‐met, probably providing evidences for developing clinical diagnosis and treatment strategies for retinoblastoma.

## 2 MATERIALS AND METHODS

### 2.1 Collection of tissue samples

Three‐hundred and fifty eyes were collected from 276 patients who received treatments in the Ophthalmology Department of the First Affiliated Hospital of Zhengzhou University from July 2015 to January 2017. The patients all did not receive chemotherapy or local radiotherapy. The retinoblastoma tissues were all pathologically diagnosed and classified by ≥2 pathologists. This study has obtained informed consents from the guardians of all patients, and it has been approved by the First Affiliated Hospital of Zhengzhou University and the ethics committee of the First Affiliated Hospital of Zhengzhou University.

### 2.2 Treatment

Post‐operative observation was conducted for patients who were hardly encroached into other tissues. Furthermore, external beam radiotherapy and/or adjuvant chemotherapy were performed for subjects who were accompanied with massive and even total choroidal invasion, who were at different degrees of lamina cribiosa, and who were encroached into glial scar, orbital tissue and sclera.

### 2.3 Cell culture

Human retinoblastoma cell lines were purchased from American Type Culture Collection (ATCC), including ACBRI‐181, Y79, HXO‐Rb44, SO‐Rb50 and WERI‐RB1. They were cultured within the purchased cell culture medium (Gibco, Grand Island, NY, USA) that contained 10% fetal bovine serum (Hyclone, South Logan, UT, USA). Moreover, the cells were cultured in 5% CO_2_ and 90% humidity at 37°C. The medium would be replaced for every 2 or 3 days.

### 2.4 Micro‐array analysis of lncRNA profile

We randomly gathered 5 pairs of retinoblastoma tissues and corresponding para‐carcinoma tissues for chip analysis (OE Biotech, Shanghai, China). To be specific, total RNA was reversely transcribed into cDNA with random primers, and hybridization was accomplished with usage of human lncRNA chip V3.0. The hybridized chips were scanned with Agilent Micro‐assay Scanner, and were analysed with aid of Agilent Feature Extraction software. The derived data were standardized and analysed utilizing Gene Spring software. The differentially expressed lncRNA was defined as one whose expression within retinoblastoma tissues was changed for at least 2 times of that within para‐carcinoma tissues, and *P* value should be <0.05.

### 2.5 Extraction of total RNA and implementation of real‐time fluorescence quantitative polymerase chain reaction (qRT‐PCR)

Total RNA was extracted from corresponding human retinoblastoma tissues and tumour cells using TRIzol reagent (Invitrogen, Carlsbad, CA, USA). The purity and concentration of RNAs were measured spectrophotometrically, and the extracted RNAs were stored at −80°C before RT‐PCR analysis. Subsequently, total RNA was subjected to reverse transcription (Invitrogen) to obtain cDNA. The reaction conditions for reverse transcription were set as: (a) 42°C for 1 hour; (b) 95°C for 5 minutes and (c) 4°C for 10 minutes. The obtained cDNA was subjected to qRT‐PCR as required by the instructions of the SYBR Green master kit (Applied Biosystems, Foster City, CA, USA). The primers (Table 1) used were designed by Primer Express software of ABI Company, and were synthesized by Shanghai Sangong Co., Ltd (Shanghai, China). The particularized reaction conditions for HOTAIR were enlisted as: (a) pre‐denaturation at 95°C for 30 seconds; (b) 40 cycles of denaturation at 95°C for 5 seconds and annealing at 60°C for 30 seconds; and (c) extension at 40°C for 5 minutes. Moreover, the reaction conditions for miR‐613 were specified as: (a) pre‐denaturation at 95°C for 10 minutes; (b) 40 cycles of denaturation at 95°C for 10 seconds and annealing at 60°C for 20 seconds; and (c) extension at 72°C for 10 seconds. The conditions for c‐met were shown as: (a) pre‐denaturation at 94°C for 1 minute, (b) 32 cycles of denaturation at 94°C for 30 seconds and annealing at 55°C for 30 seconds and (c) extension at 72°C for 2 minutes. The relative expressions of genes were calculated according to 2^−▵▵Ct^ method. GAPDH was employed as the internal control for HOTAIR and c‐met, while U6 was used as an internal reference for miR‐613.

**Table 1:** The primers for LncRNA HOTAIR, miR‐613, c‐met, U6 and GAPDH used in qRT‐PCR

Items	Primer sequence
miR‐613	5′‐GTGAGTGCGTTTCCAAGTGT‐3′ (forward)
	5′‐TGAGTGGCAAAGAAGGAACAT‐3′ (reverse)
U6	5′‐GCGCGTCGTGAAGCGTTC‐3′ (forward)
	5′‐GTGCAGGGTCCGAGGT‐3′ (reverse)
LncRNA HOTAIR	5′‐GTGGTGCTGACAAAGCTTGGAA ‐3′ (forward)
	5′‐TCACTGGGTGCCATCGTAAGAA‐3′(reverse)
c‐met	5′‐GGAGCCAAAGTCCTTTCATCTGTAA‐3′ (forward)
	5′‐GCAATGGATGATCTGGGAAATAAGAAGAAT‐3′ (reverse)
GAPDH	5′‐GGAGCGAGATCCCTCCAAAAT‐3′ (forward)
	5′‐GGCTGTTGTCATACTTCTCATGG‐3′ (reverse)

### 2.6 Cell transfection

The si‐HOTAIR, si‐NC, miR‐613 mimics, miR‐613 inhibitor and miR‐NC were all purchased from Genepharma (Shanghai, China). The recombinant plasmids of pcDNA3.1‐HOTAIR and pcDNA3.1/c‐Met were constructed with assistance of Invitrogen Life Technologies. The increased or decreased miR‐613 expression was achieved by transfection of miR‐613 mimics or miR‐613 inhibitors, while HOTAIR knockdown was achieved by transfecting si‐HOTAIR. Transfection was performed according to the instructions of Lipofectamine™ 2000 (Invitrogen), and each group of cells was harvested 24‐48 hours after transfection for further assays.

### 2.7 Dual luciferase reporter gene assay

The HOTAIR fragments that possessed specific miR‐613 binding sites were cloned into the pmirGLO dioxymase enzyme miRNA target expression vector (Promega, Madison, WI, USA) to form a reporter vector named as pmirGLO‐HOTAIR‐WT. The same XIST sequences where the miR‐34a‐5p binding sites were mutated were named as pmirGLO‐HOTAIR‐Mut. The pmirGLO‐HOTAIR‐WT or pmirGLO‐HOTAIR‐Mut were transfected with 200‐ng firefly luciferase plasmid and 4 ng of Renilla luciferase vector pRL‐TK into retinoblastoma cells that have been transfected with miR‐613 mimics or miR‐NC. Similarly, the c‐met fragment containing miR‐613 binding site was amplified by PCR, and was cloned into the pmirGLO dual luciferase expression vector to form c‐met‐wt. The specific binding site of miR‐613 within c‐met was mutated, and c‐met‐mut was produced. The cells transfected with c‐met‐wt or c‐met‐mut were, respectively, transfected with miR‐613 mimics and miR‐NC. Exactly 48 hours after transfection, luciferase activity was measured with Dual‐Luciferase Reporter Assay System (Promega).

### 2.8 Plate colony assay

The cells were inoculated within 6‐well plates at the density of 1000/well. After 8‐d culture, cell medium was discarded, and the cells were fixed with methanol. The colonies with >50 cells were calculated after staining cells with crystal violet.

### 2.9 Cell proliferation assay

After transfection for 72 hours, cells were seeded into 96‐well culture plates at a density of 2 × 10^4^/well. After 3‐4 hours when cells were adherent to tube wall, 100‐μL RPMI‐1640 complete medium and 10‐μL CCK‐8 (Dojindo Laboratories, Kumamoto, Japan) were added. The culture medium would be placed in 5% CO_2_ at 37°C for 2 hours. Microplate reader (Molecular Devices, Sunnyvale, CA, USA) was applied to measure the value of OD_450_.

### 2.10 Apoptosis assay

The apoptosis cells was detected by BD FACS Calibur flow cytometry (BD Biosciences, San Jose, CA, USA), according to the instructions of Vybrant apoptosis detection kit (Invitrogen). Within the scatter gram of bivariate flow cytometer, the lower‐left quadrant was shown as FITC‐/PI‐labelled viable cells, and the upper‐left quadrant was set as FITC−/PI + labelled viable cells. Besides, the upper‐right quadrant was presented as FITC +/PI+ ‐tagged necrosis cells, and the lower right quadrant indicated FITC +/PI‐ labelled early apoptotic cells.

### 2.11 Western blotting

The cells were lysed within RIPA buffer that contained 1% PMSF, and the proteins were loaded onto SDS‐PAGE microgels and transferred to PVDF membranes. Then 50 g/L skim milk powder was managed for blockage, and the primary antibodies diluted in 50 g/L BSA were added, including rabbit anti‐human c‐met (1: 100, Abjent, USA), mouse anti‐human E‐cadherin (1:3000, Abcam, Cambridge, MA, USA), mouse anti‐human N‐cadherin (1:8000, Abcam, Cambridge, MA), mouse anti‐human vimentin (1:1000, Cell Signaling Technology, Danvers, MA, USA) and mouse anti‐rat α‐SMA monoclonal antibody (1:300, Boster, Wuhan, China). They were placed at 4°C for overnight before incubating the blots with HRP‐conjugated goat anti‐mouse IgG (1:500, ZSGB‐BIO, Beijing, China). Darkroom visualization was achieved using ECL substrate visualization signals (Millipore, Billerica, MA, USA), and GAPDH was used as normalized endogenous white matter.

### 2.12 Statistical analysis

SPSS 17.0 software (IBM, Endicott, NY, USA) was adopted for all the statistical analyses. Kaplan‐Meier method was applied to calculate 5‐year overall survival (OS), and Log‐rank test was arranged to evaluate if OS has statistical significance. The Cox proportional hazard model was applied to compare the effects of multiple factors on OS. After conducting normal distribution test, the quantitative data (mean ± standard deviation) were analysed with usage of independent‐sample *t* test, and non‐normal distribution data were analysed using non‐parametric test analysis. The relative correlation between HOTAIR expression and miR‐613 expression within retinoblastoma tissues were assessed with Spearman correlation analysis method. Furthermore, the enumeration data were compared and analysed with chi‐square test. The Bilateral *P* values <0.05 were considered as statistically significant.

## 3 RESULTS

### 3.1 Comparison of HOTAIR and miR‐613 expressions within retinoblastoma tissues

**Figure 1:**
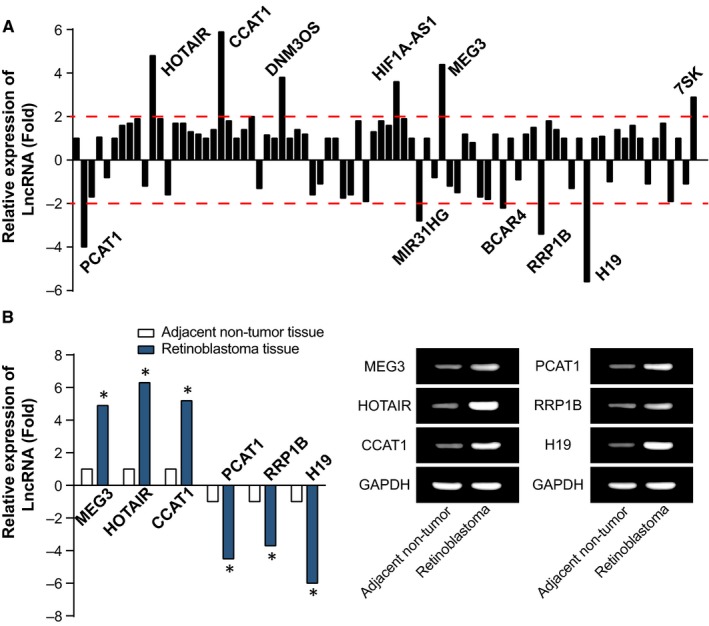
Microarray Analyses (A) for 5 Pairs of Retinoblastoma Tissues and Para‐carcinoma Tissues, and Expressions (B) of 3 Mostly Over‐expressed and Under‐expressed lncRNAs were Confirmed within the Included Retinoblastoma and Para‐carcinoma Samples. *: *P* < 0.05 when compared with adjacent non‐tumour tissues

The results of microarray analysis showed that lncRNAs HOTAIR, CCAT1, DNM3OS, HIF1A‐AS1, MEG3 and 7SK expressions were up‐regulated, and lncRNAs PCAT1, MIR31HG, BCAR4, RRP1B and H19 were down‐regulated within retinoblastoma tissues (Figure 1A). Our RT‐PCR results also verified that MEG3, HOTAIR, CCAT1 expressions within retinoblastoma tissues were far beyond those within para‐carcinoma tissues (*P* < 0.01) (Figure 1B). We chose HOTAIR for the following experiments, due to its relative stable and marked expression within retinoblastoma tissues in comparison to normal tissues.

**Figure 2:**
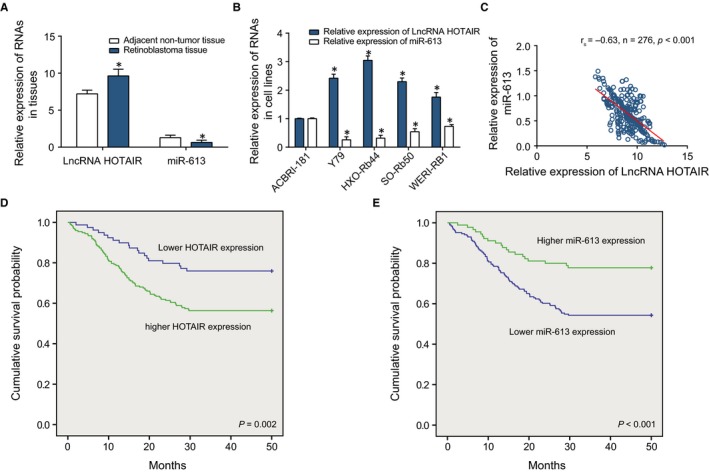
The Expressions of LncRNA HOTAIR and miR‐613 within Retinoblastoma Tissues and Cells. A, HOTAIR and miR‐613 expressions were compared between retinoblastoma tissues and adjacent non‐tumour tissues. *: *P* < 0.05 when compared with adjacent non‐tumour tissues. B, HOTAIR and miR‐613 expressions were compared among ACBRI‐181, Y79, HXO‐Rb44, SO‐Rb50 and WERI‐RB1 cell lines. *: *P* < 0.05 when compared with ACBRI‐181 cell line. C, The HOTAIR expression was negatively correlated with the miR‐613 expression within retinoblastoma tissues. D, The survival rates of retinoblastoma patients with up‐ and down‐regulated HOTAIR expressions were compared. E, The survival rates of retinoblastoma patients with highly and lowly expressed miR‐613 were compared

In addition, miR‐613 expression followed the trend opposite to HOTAIR, regarding its expression within retinoblastoma tissues (*P* < 0.05) (Figure 2A). It was also displayed that HOTAIR and miR‐613 expressions within Y79, HXO‐Rb44, SO‐Rb50 and WERI‐RB1 cell lines were profoundly different from those within ACBRI‐181 cell line (*P* < 0.05) (Figure 2B). Meanwhile, there was a negative correlation between HOTAIR and miR‐613 expressions within retinoblastoma tissues (*P* < 0.05) (Figure 2C). Furthermore, the expression of miR‐613 was evidently lower in retinoblastoma patients with tumours that were located bilaterally, poorly or non‐differentiated, larger than 10 mm, classified as T3 or T4 and were with lymph nodal metastasis (*P* < 0.05), whereas the expressions of HOTAIR was enormously higher within the above tumours than ones that were located unilaterally, well or moderately differentiated, <10 mm, classified as T1 or T2 and were without lymph nodal metastasis (*P* < 0.05) (Table 2). As the multi‐factor regression analysis showed, higher HOTAIR expression, lower miR‐613 expression, larger tumour size and grade T3 + T4 had significantly statistical relevance with the undesirable OS rate (*P* < 0.05) (Table 3) (Figure 2D‐E).

**Table 2:** The relationship between lncRNA HOTAIR/microRNA‐613 expressions and the retinoblastoma patients’ main characteristics

Main characteristics (N = 276, 350 eyes)	Long non‐coding RNA HOTAIR expression			microRNA‐613 expression		
	Low	High	*P* value	Low	High	*P* value
Mean age (months)						
<24	42	98		96	44	
≥24	37	99	0.608	90	46	0.671
Gender						
Female	38	95		83	50	
Male	41	102	0.985	103	40	0.0884
Tumor location						
Unilateral	47	155		147	55	
Bilateral	32	42	0.001	39	35	0.002
History of RB						
No	49	117		105	61	
Yes	30	80	0.686	81	29	0.072
Differentiation						
Well + moderate	83	146		137	92	
Poor + undifferentiated	28	93	0.012	88	33	0.017
Tumor size						
<10 mm	85	153		142	96	
≥10 mm	26	86	0.019	83	29	0.009
TNM classification						
T1 + T2	74	132		115	91	
T3 + T4	37	107	0.043	110	34	<0.001
Lymph nodes metastasis						
No	57	101		85	73	
Yes	22	96	0.002	101	17	<0.001

RB, retinoblastoma.

**Table 3:** The correlation between main characteristics and the retinoblastoma patients’ overall survival

Main characteristics	Univariate analysis			Multivariate analysis		
	Hazard ratio	95% CI	*P* value	Hazard ratio	95% CI	*P* value
LncRNA HOTAIR expression						
High vs low	2.44	1.35‐4.35	0.003	2.42	1.28‐4.55	0.006
miR‐613 expression						
Low vs high	2.95	1.66‐5.23	<0.001	2.69	1.44‐5.04	0.002
Mean age (months)						
<24 vs ≥24	1.26	0.77‐2.05	0.354	1.31	0.78‐2.19	0.302
Gender						
Female vs male	1.03	0.63‐1.67	0.921	1.09	0.65‐1.82	0.744
Tumor location						
Unilateral vs bilateral	1.52	0.86‐2.67	0.151	1.07	0.57‐1.99	0.837
History of RB						
Yes vs no	1.08	0.65‐1.75	0.77	0.98	0.58‐1.64	0.95
Differentiation						
Poor + undifferentiated vs Well + moderate	2.17	1.39‐3.45	0.001	1.16	0.63‐2.13	0.624
Tumor size						
≥10 mm vs <10 mm	4.27	2.63‐6.67	<0.001	5	2.86‐9.09	<0.001
TNM classification						
T3 + T4 vs T1 + T2	2.5	1.61‐3.85	<0.001	1.89	1.06‐3.33	0.031
Lymph nodes metastasis						
Yes vs No	1.67	1.02‐2.70	0.043	1.09	0.63‐1.85	0.759

CI, confidence interval; RB, retinoblastoma.

### 3.2 HOTAIR and miR‐613 regulated viability, apoptosis and EMT process of human retinoblastoma cells

**Figure 3:**
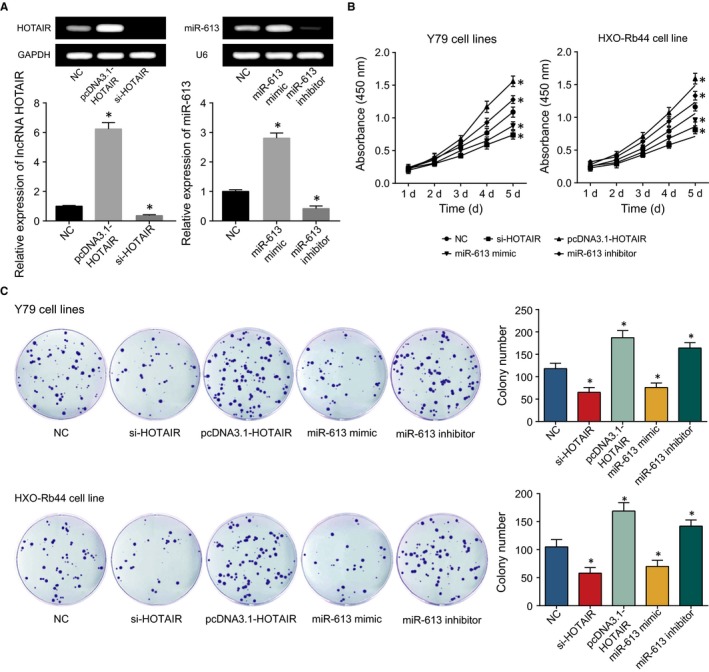
After Transfection of pcDNA3.1‐HOTAIR, si‐HOTAIR, miR‐613 mimic and miR‐613 Inhibitor into Retinoblastoma Cells (A), the Results of Colony CCK‐8 Assay (B) and Formation Assay (C) were Evaluated among the NC, si‐HOTAIR, pcDNA3.1‐HOTAIR, miR‐613 mimic and miR‐613 Inhibitor Groups. *: *P* < 0.05 when compared with NC

**Figure 4:**
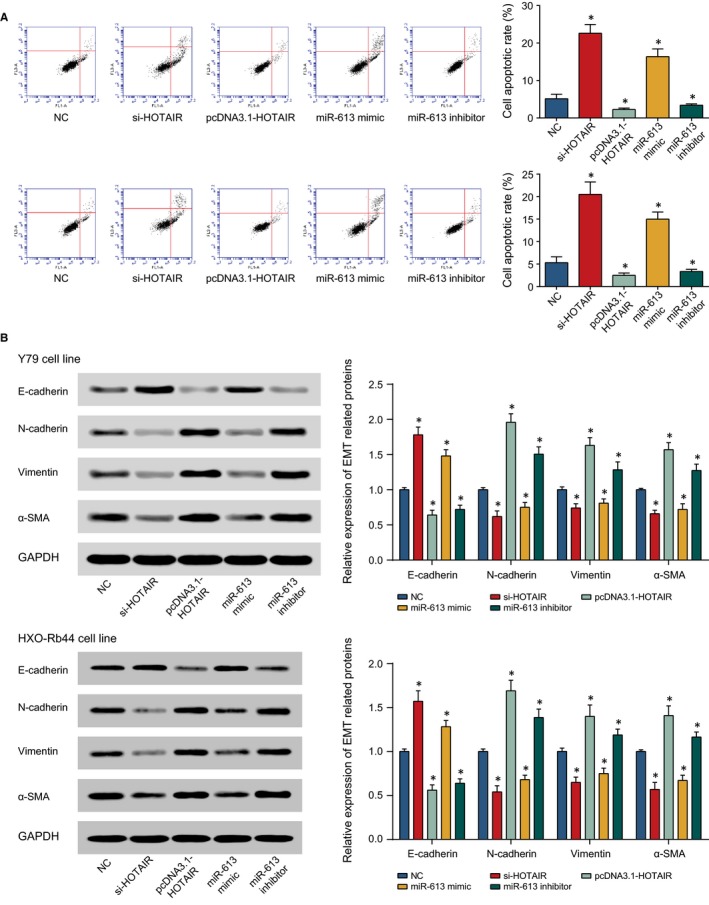
The Apoptotic Conditions (A) and Expressions of EMT Proteins (ie, E‐cadherin, N‐cadherin, vimentin and α‐SMA) (B) of Y79 and HXO‐Rb44 Cell Lines were Assessed Among Groups of NC, si‐HOTAIR, pcDNA3.1‐HOTAIR, miR‐613 mimic and miR‐613 Inhibitor. *: *P* < 0.05 when compared with NC

HOTAIR expression was up‐regulated after transfection of pcDNA3.1‐HOTAIR (*P* < 0.05), and was down‐regulated after treatment with si‐HOTAIR (*P* < 0.05) (Figure 3A). Following a similar trend, miR‐613 was highly expressed after treatment with miR‐613 mimic (*P* < 0.05), and was lowly expressed after treatment with miR‐613 inhibitor (*P* < 0.05). Moreover, when compared with NC group, the proliferative ability of Y79 and HXO‐Rb44 cells in si‐HOTAIR and miR‐613 mimic groups were conspicuously inhibited, but the capacity was remarkably improved in the pcDNA3.1‐HOTAIR and miR‐613 inhibitor groups (*P* < 0.05) (Figure 3B‐C). Conversely, the number of apoptotic cells was increased in si‐HOTAIR and miR‐613 mimics groups, while the apoptotic cells in the pcDNA3.1‐HOTAIR and miR‐613 inhibitor groups were fantastically reduced (*P* < 0.05) (Figure 4A).

Meanwhile, transfection with si‐HOTAIR and miR‐613 mimic were correlated with over‐expressed E‐cadherin, along with low‐expressed N‐cadherin, vimentin and α‐SMA. On the contrary, addition of pcDNA3.1‐HOTAIR and miR‐613 inhibitor were associated with up‐regulated N‐cadherin, vimentin and α‐SMA expressions, as well as down‐regulated E‐cadherin expression (*P* < 0.05) (Figure 4B).

### 3.3 Lnc RNA HOTAIR targeted miR‐613 to restrain its expression

**Figure 5:**
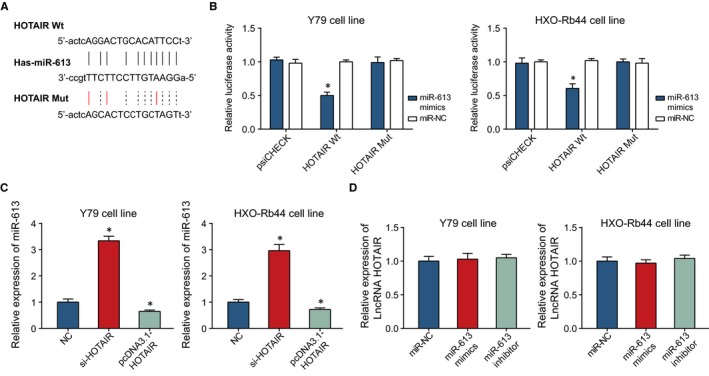
The Correlation Between HOTAIR and miR‐613. A, HOTAIR was targeted by miR‐613 in certain sites. B, The luciferase activities of Y79 and HXO‐Rb44 cell lines were compared between miR‐613 mimic + HOTAIR Wt and miR‐613 mimic + HOTAIR Mut groups. *: *P* < 0.05 when compared with miR‐NC. C, The effects of si‐HOTAIR and pcDNA3.1‐HOTAIR on miR‐613 expression were compared within Y79 and HXO‐Rb44 cell lines. *: *P* < 0.05 when compared with NC. D, The effects of miR‐613 mimic and miR‐613 inhibitor on HOTAIR expression were compared within Y79 and HXO‐Rb44 cell lines. *: *P* < 0.05 when compared with miR‐NC

To demonstrate that HOTAIR can target miR‐613, Starbase 2.0 software predicted that there were totally 29 targeting sites found for HOTAIR and miR‐613 (http://starbase.sysu.edu.cn/seedTargetInfo.php?type=lncRNA&database=hg19&name=hsa-miR-613&geneName=HOTAIR&autoId=1819&orgTable=mirLncRNAInteractionsAll). The results of dual luciferase reporter assay also displayed that the luciferase activity of cells transfected with pmirGLO‐HOTAIR‐WT and miR‐613 mimic was conspicuously lower than that of NC group (*P* < 0.05) (Figure 5A‐B). Hardly, any significant distinctions were detected between cells transfected with pmirGLO‐HOTAIR‐MUT and miR‐613 mimic and cells of NC group (*P* > 0.05).

In addition, qRT‐PCR showed that highly expressed HOTAIR greatly decreased the miR‐613 expression within Y79 and HXO‐Rb44 cells (*P* < 0.05) (Figure 5C), while the altered miR‐613 expression failed to exert any effects on HOTAIR expression (*P* > 0.05) (Figure 5D).

### 3.4 C‐MET expression was modified by HOTAIR and miR‐613

**Figure 6:**
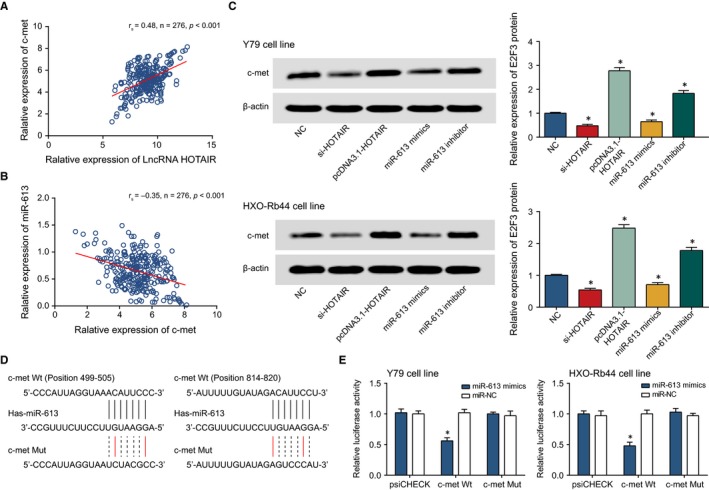
The Correlation Between miR‐613 and c‐met. A, HOTAIR expression was positively correlated with c‐met expression within retinoblastoma tissues. B, MiR‐613 expression was negatively correlated with HOTAIR expression within retinoblastoma tissues. C, The c‐met expression within Y79 and HXO‐Rb44 cell lines was compared among si‐HOTAIR, pcDNA3.1‐HOTAIR, miR‐613 inhibitor, miR‐613 mimic and NC groups. *: *P* < 0.05 when compared with NC. D, MiR‐613 was targeted by certain sites within c‐met. E, The luciferase activities of Y79 and HXO‐Rb44 cell lines were compared between miR‐613 mimic + c‐met Wt and miR‐613 mimic + c‐met Mut groups. *: *P* < 0.05 when compared with miR‐NC

HOTAIR expression was positively correlated with c‐met expression, and miR‐613 expression was negatively correlated with c‐met expression within the retinoblastoma patients (*P* < 0.05) (Figure 6A‐B). More than that, the c‐met expression was down‐regulated under the influence of lowly expressed HOTAIR or over‐expressed miR‐613, while c‐met expression was up‐regulated when miR‐613 was knocked out or when pcDNA3.1‐HOTAIR plasmid was added (*P* < 0.05) (Figure 6C) It was implied that HOTAIR promoted c‐met expression, and miR‐613 played an opposite role. It was also mirrored that the luciferase activity of miR‐613 binding to c‐met‐Wt was significantly lower than that of its binding to c‐met‐Mut (*P* < 0.05) (Figure 6D‐E), suggesting the targeted relationship between miR‐613 and c‐met.

### 3.5 HOTAIR and miR‐613 modified proliferation, apoptosis and EMT process of retinoblastoma cells via regulation of c‐met

**Figure 7:**
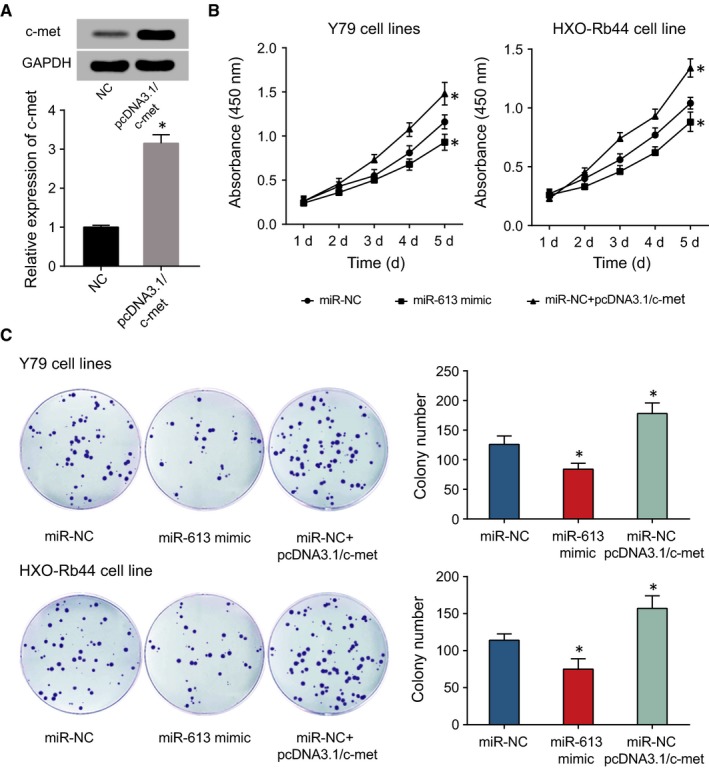
After Transfection of pcDNA3.1/c‐met into Retinoblastoma Cells (A), CCK‐8 Assay (B) and Colony Formation Assay (C) were Conducted to Compare the Proliferative Statuses of Y79 and HXO‐Rb44 Cell Lines among miR‐NC, miR‐613 mimic and miR‐NC + c‐met Groups. *: *P* < 0.05 when compared with miR‐NC or NC

**Figure 8:**
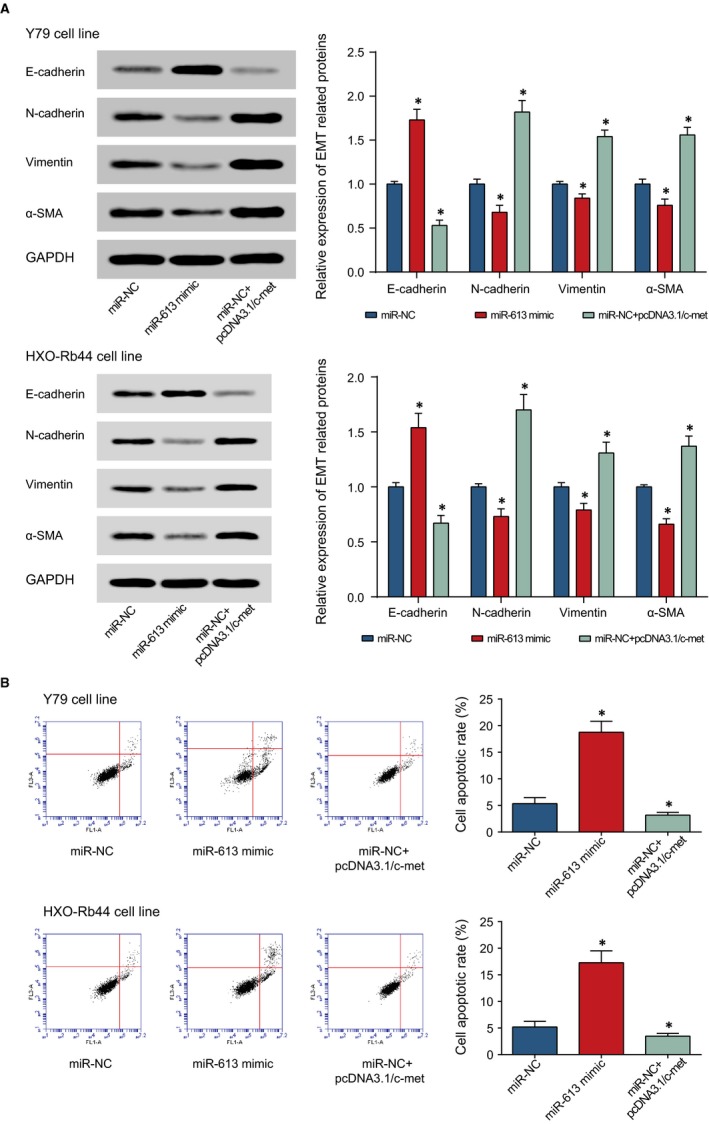
The Expressions of EMT Proteins (ie, E‐cadherin, N‐cadherin, vimentin and α‐SMA) (A) and Apoptotic Conditions (B) of Y79 and HXO‐Rb44 Cell Lines were Assessed among miR‐NC, miR‐613 mimic and miR‐NC + c‐met Groups. *: *P* < 0.05 when Compared with miR‐NC

According to Figure 7A, c‐met expression was significantly promoted in the pcDNA3.1/c‐met group, when compared with NC group (*P* < 0.05). The results of CCK8 and cell account displayed that the OD value of miR‐NC+pcDNA3.1/c‐met group was obviously higher than that of miR‐NC group (*P* < 0.05) (Figure 7B‐C), suggesting that the weakened effects induced by miR‐613 on retinoblastoma cells’ proliferation were inhibited by c‐met. Through examination of EMT‐related proteins, down‐regulated E‐cadherin and up‐regulated N‐cadherin, vimentin or α‐SMA were found in miR‐NC + pcDNA3.1/c‐met group when compared with miR‐NC group (*P* < 0.05) (Figure 8A). Flow cytometry showed that the apoptotic rate of miR‐NC + pcDNA3.1/c‐met group was markedly lower than that of miR‐NC group (*P* < 0.05) (Figure 8B), reflecting that c‐met inhibits the increased apoptosis of retinoblastoma cells induced by miR‐613. All in all, it was show that c‐met appeared as an important regulatory factor regulating the tumorigenicity of retinoblastoma cells.

## 4 DISCUSSION

Retinoblastoma, an intraocular malignancy commonly found among children, possessed a worldwide prevalence of up to 1/200 000,^32^, ^33^, ^34^ and the novel cases per year within China accounted for as high as 20% of that within the global range.^35^ Despite the emergence of diverse therapies for retinoblastoma, novel biological therapies, especially molecular targeted therapies, were still in urgent demand owing to the unneglectable toxicity and adverse reactions of current treatment strategies.

Recently, lncRNAs and miRNAs have been verified to be implicated in the onset and development of multifold neoplasms, concerning their functioning on downstream molecules.^36^, ^37^ Moreover, lncRNAs seemed to penetrate into all aspects of gene regulation, including epigenetic inheritance, transcription, post‐transcription and translation.^38^, ^39^ Interestingly, Jun Huang et al^40^ reported that lncRNA MALAT1 mediated autophagy of retinoblastoma cells through acting on miR‐124. Alternatively, lncRNA H19 inhibited progression of retinoblastoma cells via offsetting the action of miR‐17‐92 cluster,^41^ and lncRNA CCAT1 negatively regulated miR‐218‐5p to facilitate the proliferation and growth of SO‐RB50 and Y79 cell lines.^13^ HOTAIR, situated in chromosome 12 between HOXC11 and HOXC12, covered a length of 2.2 nt,^14^, ^42^ so it was also called as long intergenic non‐coding RNA (lincRNA). The lncRNA was equipped with 2 active domains, including 5′‐end that could recruit PRC2, and 3′‐end that combined with lysine specific demethylase (LSD1), RE1‐silencing transcription factor (REST) and REST co‐inhibitory protein (CoREST).^43^ It was also involved with epigenetic modulation of genes, and its interaction with polycomb repressive complex 2 (PRC2) turned as a crucial process within various cellular pathways.^44^ Our study also demonstrated that HOTAIR acted as an oncogene for promoting retinoblastoma development (Figures 3, 4).

Of note, lncRNAs could act as a competitive endogenous RNA (ceRNA) to affect miRNA expression and to influence actions of miRNA target genes, thereby modifying tumour processes, such as cell multiplication and cell apoptosis.^45^, ^46^ For example, HOTAIR could also serve as the competitive RNA for PIK3R3 to sponge miR‐214 and miR‐217, so then elevating PIK3R3 expression within ovarian cancer cells.^47^ Furthermore, HOTAIR could sponge miR‐1, miR‐214‐3p and miR‐330‐5p to raise MAPK1 expression, through which the proliferation, migration and invasion of ovarian carcinoma cells (ie, SKOV3) were largely facilitated.^48^ The present study manifested that HOTAIR sponged miR‐613 to encourage the EMT process within retinoblastoma cells (Figure 4). Interestingly, another study insinuated that miR‐613 was subjected to control of lncRNA DANCR in simultaneously acting on exacerbation of retinoblastoma.^49^ It could be explained that several lncRNAs co‐modified miR‐613 to accelerate or decelerate the aggravation of retinoblastoma.

As for miR‐613, its diminished expression seemed as a predictor for improved progression‐free survival and overall survival of ovarian cancer patients,^50^ and it also enhanced gastric cancer cells to progress by repression of brain‐derived neurotrophic factor.^51^ Our study displayed that c‐met was the downstream molecule of miR‐613, and they collaborated to modulate proliferation, viability, apoptosis and EMT process of retinoblastoma cells (Figures 6, 7, 8). Generally speaking, EMT of tumour cells occurred with fading away of inter‐cellular junctions, and incredible invasion and migration of tumour cells, so it has been deemed as a major step inherent in tumour development and migration. The previous researches also corroborated the promoting role of c‐met in EMT process within retinoblastoma. For instance, the gastric cancer cells treated with HGF (−)/c‐met (+) were accompanied with increased expressions of Snail‐2, E‐cadherin vimentin and other EMT‐relevant proteins, and cells assumed such changes as loss of polarity and increased pseudopodium of mesenchymal cells.^52^ Furthermore, c‐met also mediated epithelial‐mesenchymal transition (EMT) of breast cancer cells via activation of c‐Src and Stat3.^53^

In spite of the above‐mentioned linkage between HOTAIR/miR‐613/c‐met axis and EMT process of retinoblastoma cells, additional mechanisms were also probably inherent in the contribution of HOTAIR to development of tumours (eg, retinoblastoma). For instance, HOTAIR was previously demonstrated to alter the proliferative efficiency of neoplasms through modulating p21 expression, and p14 or p16‐dependent pathways.^54^, ^55^ It was suspected that the regulatory effects exerted by HOTAIR on tumour onset and progression differed with tumour type, so further validations should be performed through both in‐vivo and in‐vitro investigations. In addition, this investigation was limited in its incorporating sample size and not establishing models to implement in‐vivo experiments. Finally, animal models were in demand to verify the results drawn from this investigation.

Conclusively, our study results initially manifested that highly expressed HOTAIR could promote development and aggravation of retinoblastoma by miR‐613/c‐met axis, providing strong evidences for developing potential diagnostic biomarkers and treatment targets for retinoblastoma. However, further investigations were in demand to verify the consequences of this study.

## CONFLICT OF INTEREST

None.
